# Continued versus Suspended Cardiac Resynchronization Therapy after Left Ventricular Assist Device Implantation

**DOI:** 10.1038/s41598-020-59117-w

**Published:** 2020-02-13

**Authors:** Henri Roukoz, Adarsh Bhan, Ashwin Ravichandran, Mustafa M. Ahmed, Geetha Bhat, Jennifer Cowger, Munazzah Abdullah, Rahul Dhawan, Jaimin R. Trivedi, Mark S. Slaughter, Rakesh Gopinathannair

**Affiliations:** 1 University of Minnesota, Minneapolis, MN USA; 2 Advocate Christ Medical Center, Oak Lawn, IL USA; 3 St. Vincent Heart Center, Indianapolis, IN USA; 4 University of Florida, Gainesville, FL USA; 5 Henry Ford Hospital, Detroit, MI USA; 6 University of Nebraska Medical Center, Omaha, NE USA; 7 University of Louisville, Louisville, KY USA

**Keywords:** Cardiac device therapy, Outcomes research

## Abstract

Cardiac resynchronization therapy (CRT) improves outcomes in heart failure patients with wide QRS complex. However, CRT management following continuous flow Left Ventricular Assist Device (LVAD) implant vary: some centers continue CRT while others turn off the left ventricular (LV) lead at LVAD implant. We sought to study the effect of continued CRT versus turning off CRT pacing following continuous flow LVAD implantation. A comprehensive retrospective multicenter cohort of 295 patients with LVAD and pre-existing CRT was studied. CRT was programmed off after LVAD implant in 44 patients. We compared their outcomes to the rest of the cohort using univariate and multivariate models. Mean age was 60 ± 12 years, 83% were males, 52% had ischemic cardiomyopathy and 54% were destination therapy. Mean follow-up was 2.4 ± 2.0 years, and mean LVAD support time was 1.7 ± 1.4 years. Patients with CRT OFF had a higher Interagency Registry for Mechanically Assisted Circulatory Support (INTERMACS) mean profile (3.9 vs 3.3, p = 0.01), more secondary prevention indication for a defibrillator (64.9% vs 44.5%, p = 0.023), and more pre-LVAD ventricular arrhythmias (VA) (77% vs 60%, p = 0.048). There were no differences between the CRT OFF and CRT ON groups in overall mortality (Log rank p = 0.32, adjusted HR = 1.14 [0.54–2.22], p = 0.71), heart transplantation, cardiac and noncardiac mortality, all cause hospitalizations, hospitalizations for ICD shocks, and number and frequency of ICD shocks or anti-tachycardia pacing therapy. There were no differences in post LVAD atrial arrhythmias (AA) (Adjusted OR = 0.45 [0.18–1.06], p = 0.31) and ventricular arrhythmias (OR = 0.65 [0.41–1.78], p = 0.41). There was no difference in change in LVEF, LV end diastolic and end systolic diameters between the 2 groups. Our study suggests that turning off CRT pacing after LVAD implantation in patients with previous CRT pacing did not affect mortality, heart transplantation, device therapies or arrhythmia burden. A prospective study is needed to confirm these findings.

## Introduction

Cardiac resynchronization therapy (CRT) has been shown to improve mortality, left ventricular (LV) dimensions, functional status, and quality of life in patients with heart failure (HF), a left ventricular ejection fraction (LVEF) ≤35% and a wide QRS^1–4^. CRT can also decrease ventricular arrhythmias (VA) in responders and patients with LBBB^5–8^. Despite optimal therapy, some patients progress to advanced HF requiring left ventricular assist device (LVAD) support. LVADs have been shown to improve mortality, morbidity, functional status, and quality of life in advanced HF patients^9–11^. Many patients with LVAD have pre-existing CRT devices and continue to receive CRT therapy after LVAD implantation. However, the benefit of CRT in patients with LVAD remains unclear as these patients were not included in CRT trials.

Two studies showed possible decrease in VA but no overall survival benefit for CRT in patients with LVAD^12,13^. However, these single center studies are limited by a very small sample size and low power. Other studies showed no benefits in arrhythmia burden or survival^14,15^. None of these studies reported the effect of turning off CRT pacing in patient with preexisting CRT devices. Any additional benefit from CRT on survival, hospitalizations and recovery of LV function in continuous flow LVAD patients would be important to know; no benefit would solidify the decision to turn off the LV lead following LVAD implant, prolonging battery life and limiting pulse generator replacements and potential morbidity associated with the procedure.

## Methods

### Patient population

This retrospective multicenter study included 295 patients with pre-existing CRT who underwent continuous flow LVADs between 2007 and 2015 at five high volume LVAD centers in the United States (University of Louisville, Louisville, KY, University of Minnesota, Minneapolis, MN, Advocate Christ Medical Center, Oak Lawn, IL, University of Florida, Gainesville, FL, St. Vincent Hospital, Indianapolis, IN). The study protocol was approved by the Institutional Review Boards at all the centers including the University of Minnesota Institutional Review Board. All methods were carried out in accordance with relevant guidelines and regulations. Informed consent was waived by the Institutional Review Board due to the retrospective nature of the study. Patients who died during the index hospitalization for LVAD implantation, patients who underwent CRT-D implantation after LVAD implantation and patients whose CRT pacing was turned off more than 60 days after LVAD implantation were excluded from the analysis. All patients had LVADs implanted either as a bridge-to-transplantation or as destination therapy. HeartMate II^®^ (Abbott Medical, Chicago, IL) was implanted in 253 patients and Heartware^®^ (HeartWare International, Inc., Framingham, MA) in 42 patients.

The study population was divided into a CRT ON group where biventricular pacing was maintained following LVAD implant (n = 251) and a CRT OFF group (n = 44) where CRT pacing was discontinued within 60 days after LVAD implantation. The reasons for turning the LV lead off included LV lead damage during LVAD implantation, VA deemed to be driven by LV pacing and physician discretion.

### Definitions and outcomes

The data variables collected include demographics, etiology of HF, co-morbidities, LVAD type, indication (bridge to transplant vs. destination therapy) and date of implant, Interagency Registry for Mechanically Assisted Circulatory Support (INTERMACS) profile, medications, ECG and echocardiographic parameters, CRT device interrogation information including percentage of biventricular pacing, as well as incidence of ICD shocks, atrial arrhythmias (AA), and VA. The day of LVAD implant marked the start date for follow up.

We compared the CRT ON and CRT OFF groups based on the following outcomes: All cause, cardiac and non-cardiac mortality, heart transplantation, all-cause hospitalizations, heart failure and ICD therapy related hospitalizations, incidence of AA, VA, and ICD therapies including shocks and anti-tachycardia pacing (ATP). Cardiac mortality was defined as death attributable to heart failure, cardiac circulatory arrest or cardiac arrhythmias. The utilization of cardiac medications during follow-up was also reviewed to assess for any differences. Reported ECG and echocardiographic parameters during follow-up were assessed during the 6 to 12 month period post-LVAD implant. Echocardiographic data included LVEF, end diastolic and end systolic left ventricular dimensions (LVEDD and LVESD). In those patients who had less than 6 months of follow-up, the latest available information on these parameters was selected. Patient medical records as well as institutional databases at each participating center were reviewed to assess the cause of death. Whenever available, post-mortem device interrogations were reviewed to exclude an arrhythmic cause of death.

The adequacy of biventricular pacing before and after LVAD implant was confirmed by 12-lead ECG and device interrogation. The CRT devices in the CRT ON group were kept in the DDD(R) (VVIR in patients with permanent atrial fibrillation) with AV delay settings to allow consistent biventricular pacing. CRT programming was left to the discretion of the patient’s electrophysiologist and no standardized programming protocol was used. ECGs and stored device electrograms were analyzed for incidence of AA. VA and ICD therapies. VA was defined as sustained ventricular tachyarrhythmias lasting >30 sec or requiring ICD therapy (anti-tachycardia pacing or shocks). AA was defined as atrial tachycardia, atrial flutter or atrial fibrillation lasting either >6 hours or ≥1% burden on device interrogation or requiring pharmacological or electrical therapy for termination. HF hospitalization was defined as any hospitalization secondary to clinical signs and symptoms of congestive HF (dyspnea, fatigue, volume overload, as well as use of intravenous diuretics and/or inotropes for volume) and included device malfunction (LVAD thrombosis) and aortic insufficiency related HF^14^.

### Statistical analysis

Continuous variables were evaluated for normality and are shown as mean ± SD or medians [25,75] as appropriate. Categorical variables are presented as percentages. Categorical variables were analyzed using Fisher’s exact and/or Chi-square tests. Continuous variables were analyzed using non-parametric (Kruskal-Wallis) or student’s t-test as appropriate. Within groups, pre- and post-LVAD parameters were compared using paired T-tests. Kaplan-Meier Curves were used to assess survival and time dependant outcomes and the log-rank test was used to compare survival estimates. For mortality analysis, patients who underwent heart transplantation were censored in both groups. Multivariate cox regression and mixed parametric modeling were used to identify predictors of outcomes and adjust for significant differences between the 2 groups. A p-value < 0.05 was considered statistically significant. All statistical analyses were performed using the JMP Pro 14.0 (SAS Institute Inc, Cary, NC).

## Results

### Baseline characteristics

There were a total of 295 patients with LVAD implantation and pre-existing CRT device. The mean age was 60 ± 12 years and 83% of patients were males. Ischemic cardiomyopathy was the etiology of HF in 52% of patients and 54% of LVADs were implanted as destination therapy. The mean INTERMACS profile was 3.5 ± 1.4. The total mean follow-up was 2.4 ± 2.0 years. The mean LVAD support time was 1.7 ± 1.4 years for the overall cohort, 1.6 ± 1.2 years for the CRT OFF group and 1.7 ± 1.5 years for the CRT OFF group (p = 0.42).

There were 251 patients in the CRT ON group and 44 patients in the CRT OFF group. Of the 44 patients in the CRT OFF group, the reason for turning off the LV lead was because of cutting the LV during LVAD implantation in 6 patients, high LV thresholds in 14 patients, physician discretion in 9 patients (estimated to be pro-arrhythmic in 4 patients, to conserve battery life without clear high threshold in 4 patients and one patient found to have an underlying narrow QRS), phrenic nerve stimulation in 3 patients, device extraction for infection or other lead dislodgement with re-implantation of an ICD rather than a CRT device in 5 patients, and unknown in 7 patients.

The baseline characteristics including demographics, co-morbidities, echocardiographic, electrocardiographic, device related data and medical therapy are presented in Table 1. The CRT OFF group had a higher INTERMACS mean profile (3.9 ± 1.4 vs 3.3 ± 1.3, p = 0.009), more secondary prevention indication for a defibrillator (64.9% vs 44.5%, p = 0.023), less pulmonary hypertension (23.3% vs 45.8%, p = 0.007) and less systemic hypertension (52.3% vs 69.3%, p = 0.026). They also had more VA before LVAD implantation (76.9% vs 60.3%, p = 0.048). The medication use pre and post LVAD implantation was very similar with minor differences: there was more use of nitrates pre LVAD implantation, and less use of digoxin and hydralazine after LVAD implantation in the CRT OFF group. The 2 groups had similar echocardiographic and EKG parameters. In the CRT ON group, the mean biventricular pacing percentage was 96 ± 5.3%.

**Table 1:** Baseline characteristics.

	CRT ON (N = 251)	CRT OFF (N = 44)	P value
**Demographic and Medical History**			
Age (years ± SD)	60 ± 0.8	63 ± 1.8	0.10
Male (%)	82.9	84.1	0.84
Race (white, %)	71.1	61.4	0.33
BMI (mean ± SD)	29.5 ± 7.9	28.7 ± 5.4	0.47
ICM (%)	51.9	54.8	0.87
LVAD type (HM2 vs HW, %)	84.9	90.9	0.29
LVAD indication (DT vs BTT, %)	54.3	51.2	0.72
INTERMACS profile (mean ± SD)	3.3 ± 1.3	3.9 ± 1.4	**0.009**
Profile 1–2 (%)	28.1%	14.3%	
Profiles 3–7 (%)	71.9%	85.7%	**0.049**
Indication CRT-D (primary vs secondary, %)	55.5	35.1	**0.023**
COPD (%)	22.3	20.4	0.78
CAD (%)	61.3	56.8	0.57
Pulmonary HTN (%)	45.8	23.3	**0.007**
OSA (%)	35.5	38.6	0.68
PE/DVT (%)	9.2	4.6	0.39
HTN (%)	69.3	52.3	**0.026**
DM (%)	44.6	43.2	0.86
HPL (%)	69.8	59.1	0.16
CVA (%)	17.6	15.9	0.78
CKD (%)	45.0	45.5	0.96
Smoking (%)	55.4	59.1	0.65
Alcohol (%)	22.7	25.0	0.87
Mean follow-up on LVAD support (years ± SD)	1.6 ± 1.2	1.7 ± 1.5	0.42
**Arrhythmia and Device History**			
Atrial Arrhythmias (%)	65.1	62.5	0.75
AA burden (%,mean ± SD)	42.9 ± 42.8	50.0 ± 42.1	0.38
Ventricular Arrhythmias (%)	60.3	76.9	**0.048**
ICD Shocks (%)	35.7	51.3	0.07
Anti Tachycardia Pacing (%)	36.0	50.0	0.12
Biventricular Pacing before LVAD implant (%,mean ± SD)	95.8 ± 6.2	96.5 ± 4.5	0.41
**Echocardiography**			
LVEF (%, mean ± SD)	15.8 ± 5.8	16.6 ± 7.7	0.50
LVEDD (cm, mean ± SD)	7.2 ± 1.0	7.3 ± 1.2	0.40
LVESD (cm, mean ± SD)	6.5 ± 1.1	6.7 ± 1.2	0.38
**ECG Data**			
QRS duration (ms, mean ± SD)	160 ± 29	152 ± 29	0.14
QTc duration (ms, mean ± SD)	535 ± 58	555 ± 71	0.09
**Pre LVAD Medications**			
ACE inhibitor or ARB (%)	55.3	59.1	0.49
Beta Blockers (%)	82.3	81.8	0.94
Digoxin (%)	40.3	43.2	0.72
Amiodarone (%)	41.1	39.6	0.75
Other AAT (%)	7.3	6.8	0.92
Aldosterone Antagonist (%)	44.3	43.2	0.88
Thiazide (%)	12.1	9.1	0.57
Loop Diuretic (%)	86.3	95.4	0.09
Nitrates (%)	24.2	40.9	**0.021**
Hydralazine (%)	13.3	22.7	0.11
**Post LVAD Medications**			
ACE inh or ARB (%)	52.8	40.5	0.38
Beta Blockers (%)	65.1	66.7	0.84
Digoxin (%)	17.5	4.8	**0.037**
Amiodarone (%)	50.2	42.9	0.38
Other AAT (%)	10.6	9.5	0.82
Aldosterone Antagonist (%)	22.5	19.1	0.69
Thiazide (%)	3.0	7.1	0.18
Loop Diuretic (%)	66.8	80.9	0.07
Nitrates (%)	14.1	9.5	0.42
Hydralazine (%)	33.2	16.7	**0.044**

AA = atrial arrhythmias; AAT = antiarrhythmic therapy; ACE = angiotensin conversion enzyme; ARB = angiotensin receptor blocker; BMI = body mass index; BTT = bridge to transplant; CAD = coronary artery disease; COPD = chronic obstructive pulmonary disease; CKD = chronic kidney disease; CRT-D = cardiac resynchronization therapy defibrillator; CVA = cerebrovascular event; DM = diabetes mellitus; DT = destination therapy; DVT = deep venous thrombosis; HM2 = HeartMate 2; HW = Heartware; HPL = hyperlipidemia; HTN = hypertension; ICD = implantable cardioverter defibrillator; ICM = ischemic cardiomyopathy;; INTERMACS = Interagency Registry for Mechanically Assisted Circulatory Support; LVAD = left ventricular assist device; LVEF = left ventricular ejection fraction; LVEDD = left ventricular end diastolic dimension; LVESD = left ventricular end systolic dimension; OSA = Obstructive sleep apnea; PE = pulmonary embolism.

### Mortality and heart transplantation

**Figure 1:**
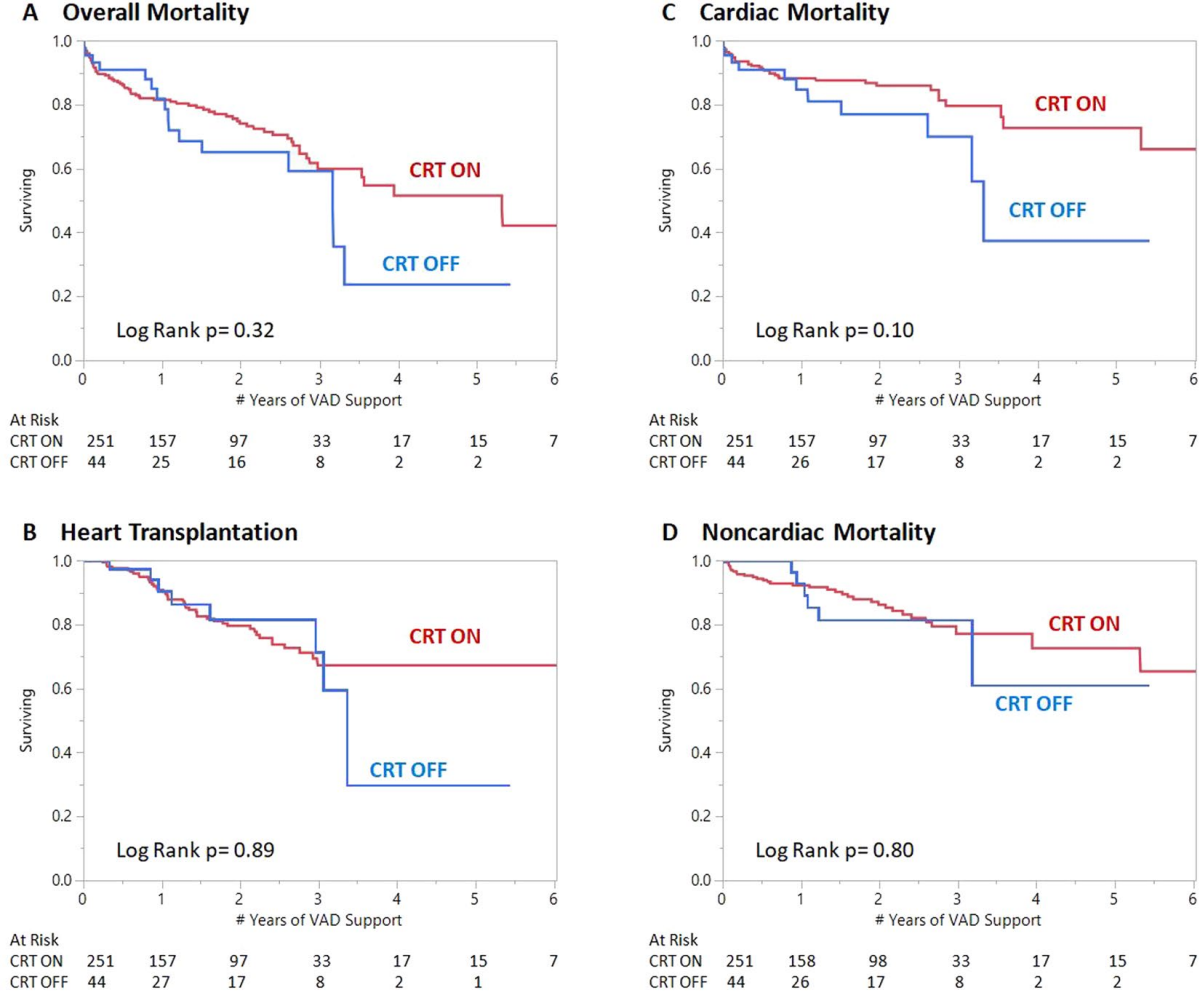
Kaplan Meier curves (unadjusted) of mortality and heart transplantation in the CRT ON = group versus CRT OFF group. Log rank test p values are presented in the bottom right of each diagram. (**A**) Overall mortality over the entire follow-up period. (**B**) Heart transplantation. (**C**) Cardiac mortality. (**D**) Noncardiac mortality.

The overall mortality during follow-up was 31.8% in the CRT ON group and 36.4% in the CRT OFF group. The Kaplan Meier curves for all-cause mortality, heart transplantation and the combined mortality or heart transplantation are presented in Fig. 1. The hazard ratios were first adjusted for the differences between the CRT ON and CRT OFF groups: pre-LVAD VA, INTERMACS profile, pulmonary hypertension, hypertension and indication for ICD. All-cause mortality was not different between the 2 groups (Log rank p = 0.32, unadjusted HR = 1.33 [0.75–2.23], p = 0.32; adjusted HR = 1.14 [0.54–2.22], p = 0.71). When adjusted for predictors of mortality in the univariate model (Age, cardiomyopathy type, destination therapy and INTERMACS profile), there was still no difference between the 2 groups (adjusted HR = 1.10 [0.53–2.13], p = 0.78).

There were no statistically significant differences in cardiac (Log Rank p = 0.10, adjusted HR = 1.25 [0.54–2.65], p = 0.58) and non-cardiac mortality (Log Rank p = 0.80, adjusted HR = 0.59 [0.16–1.62], p = 0.33). There were 132 patients who received the LVAD as a bridge to transplant, with a survival of 72.7%. There was also no statistically significant difference in the occurrence of heart transplantation between the CRT ON and CRT OFF groups (Log Rank p = 0.89, adjusted HR = 1.03 [0.39–2.42], p = 0.95). Survival outcomes are presented in Table 2.

**Table 2:** Clinical Outcomes.

	CRT ON N = 251	CRT OFF N = 44	P value	Adjusted HR or OR [95% CI], p value^a^
**Mortality and Heart Transplantation**				
Overall mortality, n(%)	75 (29.9)	16 (36.3)	0.37	1.14 [0.54–2.22], p = 0.71
OHT, n(%)	45 (18.0)	8 (18.2)	0.98	1.03 [0.39–2.42], p = 0.95
Cardiac Mortality, n(%)	37 (14.7)	10 (22.7)	0.16	1.25 [0.54–2.65], p = 0.58
Noncardiac Mortality, n(%)	38 (15.1)	6 (13.6)	0.73	0.59 [0.16–1.62], p = 0.33
**Hospitalizations**				
All cause hospitalizations	3.8 ± 4.4	3.8 ± 3.0	0.88	
All cause hospitalizations, yearly	2.3 ± 2.7	5.7 ± 15.4	0.18	0.66 [0.30–1.47], p = 0.31
Hospitalizations for ICD shock	0.2 ± 0.6	0.4 ± 0.9	0.08	
Hospitalizations for ICD shock, yearly	0.1 ± 0.5	0.5 ± 1.6	0.14	0.91 [0.82–1.02], p = 0.08
**Arrhythmias**				
ICD shock after LVAD, %	35.8	45.4	0.23	0.89 [0.39–2.1], p = 0.80
No. of ICD shocks after LVAD	3.6 ± 18.5	3.9 ± 7.1	0.83	
ATP after LVAD, %	46.1	51.2	0.56	1.14 [0.47–2.73], p = 0.77
No. of ATP after LVAD	12.3 ± 52.5	60.1 ± 286	0.28	
Post-LVAD AA, %	58.3	76.7	**0.023**	0.45 [0.18–1.06], p = 0.31
Post-LVAD VA, %	58.1	77.3	**0.018**	0.65 [0.41–1.78], p = 0.41
**Echocardiography post LVAD**				
LVEF (%, mean ± SD)	19.7 ± 12.0	16.0 ± 5.8	**0.005**	
LVEF Mean Difference (%, mean ± SD)^b^	3.8 ± 0.9	0.75 ± 1.5	0.18	
LVEDD (cm, mean ± SD)	6.1 ± 1.3	7.4 ± 1.4	0.63	
LVEDD Mean Difference (cm, mean ± SD)^b^	−1.0 ± 0.08	−1.0 ± 0.17	0.81	
LVESD (cm, mean ± SD)	5.6 ± 1.6	5.6 ± 1.1	0.78	
LVESD Mean Difference (cm, mean ± SD)^b^	−0.3 ± 0.1	−0.4 ± 0.2	0.80	

^a^The adjustments were made for differences in the two groups: pre-LVAD VA, INTERMACS profile, pulmonary hypertension, hypertension and indication for ICD. ^b^The mean difference is between pre-LVAD and post-LVAD values. AA = atrial arrhythmias; ATP = anti-tachycardia pacing; CRT = cardiac resynchronization therapy; HR = hazard ratio; ICD = implantable cardioverter defibrillator; LVAD = left ventricular assist device; LVEF = left ventricular ejection fraction; LVEDD = left ventricular end diastolic dimension; LVESD = left ventricular end systolic dimension; OHT = orthotopic heart transplantation; VA = ventricular arrhythmias.

### Hospitalizations

All cause hospitalizations and hospitalizations due to ICD therapy did not differ significantly between the two groups. The hospitalization data during the follow-up period and yearly averages is presented in Table 2.

### Arrhythmias

There was no statistically significant difference in ICD shocks or ATP incidence or burden between the 2 groups (Table 2). However, there was an increase in post LVAD VA incidence in the CRT OFF group (77% vs 58%, p = 0.018). When adjusted for pre LVAD VA, CRT pacing had no effect on post LVAD VA (OR = 0.49 [0.22–1.11], p = 0.09). When adjusted for all group differences (pre-LVAD VA, INTERMACS profile, pulmonary hypertension, hypertension and indication for ICD), there were still no differences (OR = 0.65 [0.41–1.78], p = 0.41), with the INTERMACS profile (OR = 0.21 [0.04–0.99]. p = 0.049) and pre LVAD VA (OR = 8.4 [3.72–20.6], p < 0.0001) being the main independent risk factors. The CRT OFF group also had an increased prevalence of post LVAD AA (77% vs 58%, p = 0.023). Again, when adjusted for group differences, there was no statistically significant differences between the two groups (OR = 0.45 [0.18–1.06], p = 0.31), with INTERMACS profile (OR = 0.85 [0.75–0.97], p = 0.012) and pulmonary HTN (OR = 3.12 [1.56–6.32], p < 0.0012) being independent risk factors.

### Echocardiographic parameters

There was no difference in LVESD and LVEDD between the 2 groups. There was a small but statistically significant difference in mean post LVEF between the 2 groups. However, when we compared changed in LVEF, LVEDD and LVESD from pre to post LVAD in a paired fashion, there were no statistically significant differences. Echocardiographic data is presented in Table 2.

## Discussion

This multicenter study shows that turning CRT off after LVAD implantation in patients with preexisting CRT device does not affect all cause mortality, cardiac mortality, heart transplantation, all cause hospitalizations, AA and VA or ICD therapies. There was a small but statistically significant difference in LVEF, which was higher in the CRT ON group.

Many patients undergoing LVAD implantation have a pre-existing CRT device. Turning off CRT pacing might decrease the need for generator changes and could potentially reduce the risk of infection. One study did not show any difference in generator changes performed with turning off the LV lead, with only one patient requiring multiple changes due to high LV lead thresholds^12^. However, a more recent study showed an increased chance of generator changes in LVAD patients with CRT-D compared to patients with ICD only (26% vs 15.5%)^14^. The patients who have a high LV lead pacing threshold probably would benefit the most with turning off the LV lead from generator change standpoint. Whether turning off the LV lead in all these patients will significantly impact the longevity of the CRT devices is still to be proven. Moreover, we still don’t know whether there is a need to revise or replace the LV lead in this scenario. Our study suggests that the benefit in these patients is very limited.

Currently, there is no consensus regarding the efficacy of CRT pacing in patients undergoing LVAD implantation. The studies aimed at addressing this issue are few, limited by the number of patients, or comparing patients with CRT to patients who have ICDs^12–14,16,17^. Gopinathannair *et al*. compared LVAD patients with CRT-D with patients who had ICD alone. In agreement with our study, they found no difference in mortality, cardiac and noncardiac hospitalizations, heart transplantation, LV dimensions, ICD shocks, AA or VA^17^. A larger multicenter study on the same patient population also showed no differences in these major outcomes^14^. These reports did not compare the effect of turning off CRT pacing in a preexisting device and are limited by the fact that patients with ICD only might not have had an indication for CRT pacing before LVAD implantation. Finally, a more recent study showed no acute hemodynamic changes between the intrinsic rhythm, RV pacing and CRT pacing in patients with LVAD and pre-existing CRT^18^. However, this study did not include long term follow-up.

Our report represents the largest and first multicenter study to assess the effect of turning off CRT pacing in patients around the time of LVAD implantation. Both Schleifer *et al*. (65 patients) and Richardson *et al*. (41 patients in CRT on and off arms) studied the effect of turning off CRT in patients undergoing LVAD implantation in a nonrandomized and randomized fashion, respectively^12,13^. In accordance with our study, Schleifer *et al*. showed no difference in mortality or heart transplantation. Richardson *et al*. did not report these outcomes in the CRT groups but the event rate was low. In agreement with our study, both showed no difference in hospitalizations. Schleifer *et al*. showed an increase in ICD shocks and a trend towards increase in cumulative VA, and Richardson *et al*. showed only a nonsignificant trend of increase in ICD shocks. The unadjusted data from our study also showed an increase in VA after turning CRT off, but the effect disappeared after adjusting for pre LVAD VA, and there was no difference in ICD therapies. Schleifer *et al*. did not have a difference in pre LVAD VA between the CRT ON and CRT OFF groups, but similar to our study, pre LVAD VA was the best predictor of post LVAD VA.

Our study found a higher incidence of AA in the CRT OFF group after LVAD implantation, however, after adjustment to differences between the groups, the difference in atrial arrhythmias was not statistically different with the INTERMACS profile being one of the independent risk factors. The adjusted result is in agreement with previous studies^14,17^. This observation could be due to the fact that the LVAD patients who had their CRT pacing turned off had more history of hypertension, were sicker based on their INTERMACS profile and had more complicated procedures which could lead to higher incidence of post-operative atrial fibrillation.

While it is controversial whether LVEF and LV dimension preservation is worthwhile after LVAD, our study showed that the change in LVEF, LVEDD and LVESD was not statistically different between the CRT ON and CRT OFF groups. The change in these parameters were not reported in the studies mentioned above.

Our study is limited by its retrospective and nonrandomized nature. Due to limitation in data collection from outside centers other than the ones involved in this study, full adjustment for known correlates of LVAD mortality (e.g. concomitant procedures) was not possible. Adjusted analyses cannot correct for unforeseen mortality risk factors. CRT programming could not be controlled for due to the complexity and wide variability in programmed settings driven by the lack of consensus. CRT pacing was turned off for a variety of reasons and therefore was difficult to adjust for. Furthermore, the conclusions of our study might only apply for patients with similar indications to turn off CRT pacing. However, the study does offer insights to consider in all comers.

## Conclusion

This multicenter study shows that turning CRT off after LVAD implantation in patients with preexisting CRT device does not adversely impact survival, heart transplantation or arrhythmia burden^19^. While the data herein supports the decision to inactivate biventricular pacing, a large, prospective, randomized study is needed to truly confirm these findings.
